# A Multi-Label Learning Framework for Drug Repurposing

**DOI:** 10.3390/pharmaceutics11090466

**Published:** 2019-09-09

**Authors:** Suyu Mei, Kun Zhang

**Affiliations:** 1 Software College, Shenyang Normal University, Shenyang 110034, China; 2 Bioinformatics Core of Xavier NIH RCMI Cancer Research Center, Department of Computer Science, Xavier University of Louisiana, New Orleans, LA 70125, USA

**Keywords:** drug-target interaction, drug repurposing, drug-disease associations, multi-label learning, stratified multi-label cross validation

## Abstract

Drug repurposing plays an important role in screening old drugs for new therapeutic efficacy. The existing methods commonly treat prediction of drug-target interaction as a problem of binary classification, in which a large number of randomly sampled drug-target pairs accounting for over 50% of the entire training dataset are necessarily required. Such a large number of negative examples that do not come from experimental observations inevitably decrease the credibility of predictions. In this study, we propose a multi-label learning framework to find new uses for old drugs and discover new drugs for known target genes. In the framework, each drug is treated as a class label and its target genes are treated as the class-specific training data to train a supervised learning model of l_2_-regularized logistic regression. As such, the inter-drug associations are explicitly modelled into the framework and all the class-specific training data come from experimental observations. In addition, the data constraint is less demanding, for instance, the chemical substructures of a drug are no longer needed and the novel target genes are inferred only from the underlying patterns of the known genes targeted by the drug. Stratified multi-label cross-validation shows that 84.9% of known target genes have at least one drug correctly recognized, and the proposed framework correctly recognizes 86.73% of the independent test drug-target interactions (DTIs) from DrugBank. These results show that the proposed framework could generalize well in the large drug/class space without the information of drug chemical structures and target protein structures. Furthermore, we use the trained model to predict new drugs for the known target genes, identify new genes for the old drugs, and infer new associations between old drugs and new disease phenotypes via the OMIM database. Gene ontology (GO) enrichment analyses and the disease associations reported in recent literature provide supporting evidences to the computational results, which potentially shed light on new clinical therapies for new and/or old disease phenotypes.

## 1. Introduction

Drug repurposing develops new uses for the existing or abandoned drugs to accelerate the process of drug discovery and decrease the development cost. The dogma of traditional drug discovery is primarily to seek the most specific drugs to act on specific targets for specific diseases, i.e., the paradigm of one drug-one target-one disease [1]. As a result, the progress of drug discovery via trial and error is very slow and costly. Under the therapeutic concept of “one drug multiple targets”, polypharmacology has opened a new avenue to rational development of more effective but less toxic therapeutic agents in recent years [2,3,4]. Nowadays, drug combination and drug repurposing have become effective approaches to polypharmacological drug discovery for two reasons. On one hand, a disease phenotype is often associated with multiple disease genes, urging us to develop a therapeutic policy of drug combination to increase drug efficacy [5,6]; on the other hand, a drug molecule often targets multiple target genes [7], implicating that an old drug could be reused as a therapy for new disease, i.e., drug repurposing [8]. No matter which approach we adopt, experimental identification or in silico prediction of drug-target interaction for new usage of known drugs promises to play a significant role in polypharmacological drug discovery [9].

In the last decade, many computational methods have been proposed to predict drug-target interaction and have been comprehensively reviewed. Interested readers are referred to [10,11,12,13,14] for more details about databases, web servers, and methods. In general, these methods are divided into two categories, i.e., graph-based inference and drug structure-based methods. Graph-based inference is much less demanding on scarce data and only needs the topology of a drug-target bipartite graph. For instance, Lu et al. [15] adapt the notion of common neighbors in social networks to a biological bipartite graph, by which to measure the similarity between drugs and targets. Comparatively, drug structure-based methods are more demanding on scarce data, e.g., direct information or indirect descriptor of drug structures. Especially, the docking approach additionally needs 3D structures of ligand targets [16]. Except the docking approach, the other drug structure-based methods can be further classified into network-based [12,17,18] and machine learning methods [19,20,21,22,23,24,25,26,27]. In fact, the distinction between these two types of methods is not so clear. For instance, the methods based on matrix representation of drug-target interaction (DTI) networks and inference are also categorized into machine learning methods [13,14]. In this study, to narrow the scope and facilitate discussions, machine learning methods only refer to the methods that represent DTI networks via vectorization and similarity-based kernelization. The methods based on matrix representation and inference, e.g., Laplacian regularized least squares, kernelized Bayesian matrix factorization, the bipartite local method, etc., as reviewed in [13], are categorized into networks-based methods. The methods based on DTI network topology and similarities are also categorized into networks-based methods. The network-based methods use the drug, target, disease, or network similarities to predict drug-target interaction and drug repurposing. For instance, Luo et al. [17] predict drug repurposing via random walks on networks of heterogeneous similarities. In the network-based methods, the potential noise and the very incomplete drug-target interaction (DTI) networks heavily restrict the inference of new interactions. Comparatively, machine learning methods are more attractive because they could be based on a small number of training data to resist noise and well generalize to unseen examples [28]. We discuss the existing machine learning methods from two major aspects, namely feature representation and class space of classification.

According to the feature representation of drugs and target genes, the machine learning methods are categorized into kernel representation, vector representation, and deep neural network representation. Kernel methods [19,20,21,22] implicitly embed drug structural similarity and protein similarity into kernel matrices without explicit feature representation, wherein the chemical structural similarity between drugs could be calculated via specific tools, such as SIMCOMP [29]. Kernel representation is especially useful when the information of similarities between examples are easily available. For prediction of drug-target interaction, the chemoinformatic tools used to calculate the similarities between drug structures directly determine the success and failure of modeling. Unlike kernel methods, vector representation methods [23,24,25,26,27] have to explicitly extract features or descriptors from drug and protein structures via chemoinformatic tools, such as the Rcpi package [30], to further represent drug-target pairs into flat feature vectors. Computational extraction of features from drug chemical structures has been comprehensively reviewed in [11]. As regards to target proteins, the features of sequence feature, domain, and gene ontology are also frequently used [11]. Similarly, the chemoinformatic tools used to extract features from drug structures directly affect the model performance. Deep learning is well-known for its ability of automatically embedding feature information into multiple hidden layers of neural network representations. For this reason, deep learning has been used to extract features from target protein sequences for drug-target interaction prediction [27,31,32]. A significant breakthrough in the tremendous parameter tuning and the mathematical theory behind it is urgently needed for this promising technique, improper use of which is prone to lead to overfitting.

According to the class space of classification, the existing machine learning methods are all categorized into binary classifications. The local models [24,25] train multiple binary models, with one model corresponding to one drug, and these binary models are independent without considering the inter-class or inter-drug associations. All the other machine learning methods train one global binary model, using all the known interacting pairs as positive training data and using randomly sampled drug-target pairs as negative training data. In these methods, drug-target pairs are generally represented with drug and protein structures. A perfect match at the interface between drug chemical structure and target protein structure indicates a good drug-target binding affinity, such that the structural feature representation of drug-target pairs is well interpreted in biological terms and promises to achieve good performance. However, the binary models generally require a large number of randomly sampled negative data, equivalent to or much larger than the positive data in size, such that these methods run a high risk of false negative sampling and bias. For this reason, several studies focus on improving the quality of sampled negative data [25,26]. Ezzat et al. [25] use *k*-means clustering and ensemble learning techniques to sample representative negative examples. Liu et al. [26] integrate chemical structures, chemical expression profiles, side effects of compounds, amino acid sequences, protein–protein interaction network, and gene ontology (GO) annotations of proteins to screen negative data. Although these binary classification methods have demonstrated its efficacy in predicting drug-target interaction, there are several major concerns to be addressed. First, it is oversimplified to model the huge drug space, target space, and their complex associations into binary classification without explicitly considering the inter-drug associations. Second, the involved target proteins are mostly limited to the four types of enzyme, ion channel, GPCR, and nuclear receptor. Third, equal-size negative data are required to train a binary classification model that tends to introduce more noise. Fourth, the models heavily depend on chemical structures of drugs, which are not always available. Lastly, the binary classification methods easily capture the latent patterns of the interface between drug chemical structures and target protein structures, and thus easily infer direct drug-target interactions but at the same time miss predicting indirect drug-target interactions.

In this study, we treat prediction of drug-target interaction as a problem of multi-class classification, where each drug is viewed as a class label and the genes/proteins of the drug targets are viewed as the class-specific training data. As a gene is potentially targeted by more than one drug, the multi-class classification is then converted to a problem of multi-label learning. In the proposed multi-label learning framework, we could predict new drugs for a known disease gene and at the same time predict new target genes for a known drug (i.e., drug repurposing). Since drugs are viewed as class labels, the chemical structures of drugs are no longer needed, and novel target genes of a drug are inferred only from the patterns of the known genes that the drug targets. We use gene ontology to depict the genes that drugs act on because GO terms could reveal the information of drugs about where to act (subcellular components), what to act (molecular functions), and how to act (biological processes). Lastly, we used the model to predict new drugs for all the known target genes, and further associate these new drugs with disease phenotypes via the OMIM database [5] to repurpose these drugs.

## 2. Data and Methods

### 2.1. Flowchart Overview

**Figure 1:**
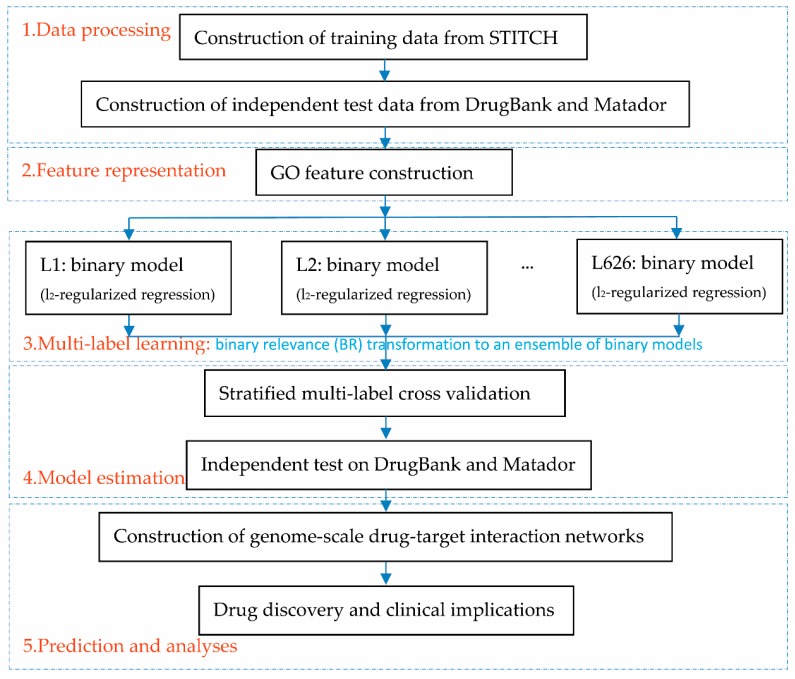
Flowchart overview of multi-label learning framework.

To help the proposed framework be easily understood, we first provide the flowchart overview, as illustrated in Figure 1. The first step prepared data, including the training data extracted from STITCH [33] and the independent test data extracted from DrugBank [7] and Matador [34]. The second step represented the target genes into binary feature vectors that indicate the presence or absence of GO terms. The third step solved the multi-label learning problem via binary relevance (BR) transformation [35], which yields the final predicted label set through an ensemble of binary models. The fourth step is to estimate the proposed framework via a stratified multi-label cross-validation and independent test. The fifth step constructed genome-scale drug-target interaction networks for drug discovery and clinical analyses.

### 2.2. Data

**Figure 2:**
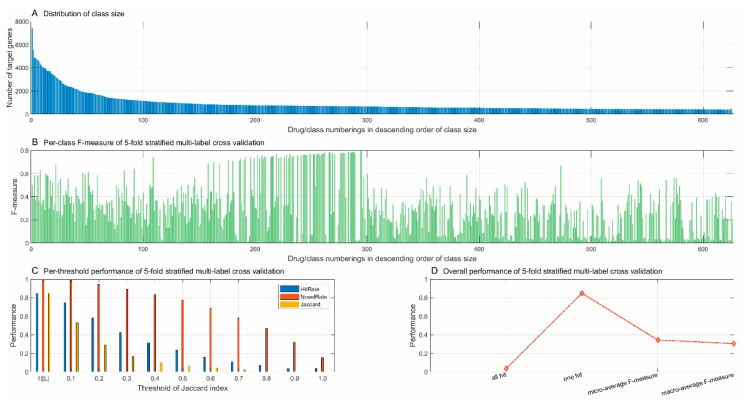
Class size distribution of training data (**A**) and performance of 5-fold stratified multi-label cross-validation (**B**–**D**).

The training data were extracted from STITCH [33]. To the best of our knowledge, STITCH [33] curates the largest number of drug-target interactions (DTI) among the major DTI databases. There were 15,473,939 drug-target interactions between 787,039 drugs and 17,501 genes. STITCH also provides confidence scores to each drug-target interaction that comes from experiments, other databases, literature, and inference. The DTI data from STITCH were subjected to the following steps of processing. First, we only chose the well-studied target genes that had been annotated with at least one GO term of molecular function (except the generic root GO term GO:0003674) or biological process (except the generic root GO term GO:0008150) to avoid null feature vectors, because gene ontology is used to represent drug-targeted genes (see the subsection “Feature construction”). Second, the drugs were treated as class labels, and the drug-targeted genes were treated as the class-specific training data. The number of targeted genes among drugs is highly imbalanced among classes. For instance, drug CIDm00003121 (valproic acid, molecular formula C_8_H_16_O_2_) contains 7398 well-studied target genes, while 78.90% of drugs are found to target fewer than 10 genes. To reduce the class space and the risk of model bias, we only chose those drugs that target more than 400 genes as class labels. The other drugs were merged as the class “others” and the training data of this class were randomly sampled from the target genes of these drugs, which were disjointed with the target genes of the drugs that were chosen as class labels. Last, to consider the case of a drug-target pair that does not interact, we added a class called the “non-target” to the class space, whose training data were randomly sampled from the well-studied human genes that were disjointed with the target genes of the chosen class labels and the class “others”. As results, we obtained in total 626 classes, including 624 chosen drugs, the class “others”, and “non-target”. The training data contained 551,673 drug-target interactions between 624 drugs and 17,176 target proteins. The class “others” and “non-target” contained 504 and 128 genes, respectively. For the convenience of illustration, the classes were consecutively numbered from 1 to 626 with the classes “others” and “non-target”, numbered 625 and 626, respectively. All the other classes were numbered in descending order of class size. The distribution of class size is illustrated in Figure 2A.

The independent test data were extracted from DrugBank [7] and Matador [34]. In these databases, drugs were named differently. We used the PubChem database [36] to map the drug space of DrugBank and Matador onto that of STITCH. DrugBank [7] is a comprehensive repository of drug-target interactions that contains the information of drugs, target genes, synergistic and adverse effects of drug-drug interactions, etc. Comparatively, Matador [34] is a small database that curates both direct and indirect drug-target interactions. From DrugBank, we extracted 27,200 drug-target interactions between 7705 drugs and 4230 genes. From Matador, we extracted 3064 drug-target interactions between 396 drugs and 2231 genes. After discarding the drug-target interactions without PubChem drug mappings, narrowing the choice to well-studied genes and further excluding the genes that have occurred in the training data, we obtained 113 drug-target interactions between 18 drugs and 82 well-studied target genes from DrugBank and obtained 482 drug-target interactions between 9 drugs and 405 well-studied target genes from Matador. As a result, the target genes in the independent test data were disjointed with those in the training data. The independent test was conducted to check how well the proposed framework correctly recognizes the known drugs of the given genes.

The prediction set came from all the well-studied target genes from STITCH, DrugBank, and Matador, amounting to 16,776, 2351 and 2045 for STITCH, DrugBank and Matador, respectively. From the predictions, we could obtain new drugs for these genes, as well as new target genes for the known drugs.

### 2.3. Multi-Label Learning Framework

In this study, we treated each drug as a class label and its target genes as the associated training data. The scenario that a gene is targeted by multiple drugs is hereto tailored to multi-label learning. In the machine learning field, two types of problem transformation, i.e., label powerset (LP) and binary relevance (BR) [35], are frequently used to convert multi-label learning to traditional multi-class learning or two-class learning. The LP transformation method treats all the label combinations as class labels, so that an exponentially large class space is yielded for a problem with a large number of classes. Assuming that *N* drugs are chosen as the class labels L={i|i=1,2,…,N}, the label space is as large as $\sum_{i=1}^{N}C_{{}_{N}}^{i}=2^{N}-1$. Furthermore, the LP transformation method potentially encounters many odd label combinations that possibly possess very few or even only one example. In such cases, class imbalance becomes very serious. Oversampling and undersampling are two major methods to tackle class imbalance in the machine learning field [37]. Oversampling randomly samples replicate examples from minority classes, while undersampling randomly discards examples from majority classes. However, the oversampling method is prone to overfitting and the undersampling method potentially results in information loss.

To reduce the class space and avoid extreme class imbalance, we choose the BR transformation method to implement the proposed multi-label learning framework. The BR transformation method actually trains an ensemble of *N*-independent binary models for *N* classes, in which each binary model is trained for a class. Formally, for each drug *i*, we used its gene set $G_{i},i=1,2,\ldots ,N$ as the positive training data and the remaining genes ($\overset{N}{\underset{j=1}{\cup }}G_{j}-G_{i}$) as the negative training data to train a binary model. For a candidate gene *g*, the BR transformation method yields a predicted label set $L^{\prime }=\{i|f_{i}(g)>0,i=1,2,\ldots N\}$. In the BR transformation method, the problem of class imbalance was still very serious with the ratio of negative to positive equal to $\left|\overset{N}{\underset{j=1}{\cup }}G_{j}-G_{i}\right|/\left|G_{i}\right|$. Fortunately, the negative class was much larger than the positive class so that the false positive rate was expected to be much lower than the false negative rate. As such, the positive labels predicted by the BR transformation method was convincingly credible. In this sense, the class imbalance helped increases the credibility of positive predictions. As compared to the LP transformation method, the BR transformation method greatly reduces the dimensionality of class space and, to a large extent, relieves the stress of class imbalance.

It is worth noting that the negative data for drug *i* (i.e., the *i*-th binary model) in the proposed framework refers to the genes that are targeted by the drugs other than drug *i*, and these data still come from experimentally observed drug-target interactions. However, the negative data used by the existing machine learning methods are randomly sampled drug-target pairs, which are assumed not to interact.

### 2.4. Feature Construction

We depict the targeted genes/proteins using GO terms from the GOA database [38]. There are three major concerns about using GO terms to represent genes/proteins, namely semantic interdependence, GO sparsity, and GO imbalance. In this study, we simply represent each gene with a binary vector denoting the presence or absence of GO terms to address the three concerns. First, although GO terms are hierarchically organized in a directed acyclic graph (DAG), we do not explicitly consider the interdependence and semantic similarities between GO terms in order to not introduce inter-feature correlations into the feature representation. The information of GO semantic similarities is more properly embedded into kernel matrices of kernel methods. Second, the solution to GO sparsity ultimately depends on the accumulation of knowledge about genes and gene products. In this study, we only choose those well-studied genes to ensure the quality of training data. Lastly, the present GO annotations are very unbalanced among genes. Some genes are overly annotated, and some GO terms (e.g., cell cycle, transcription, etc.) are richly branched with deep trees of descendant GO terms in GO DAG, while some other genes are sparsely annotated with coarse-grained GO terms. Shrinkage to an upper level of GO terms could surely do justice to all genes and, to some extent, counteract GO imbalance, but discarding a lower level of GO terms would result in information loss. Similarly, the solution to GO imbalance also ultimately depends on the constant update of knowledge about genes and gene products. Fortunately, GOA [38] is constantly kept updated, which, to some extent, relieves the stress of GO sparsity and imbalance. At present, the GO terms we use to represent genes are directly photocopied from the GOA database, such that the updates of GOA could be conveniently incorporated into the binary feature vectors of this proposed framework. It is worth noting that some other databases, such as Reactome (https://reactome.org/), WikiPathways (https://www.wikipathways.org/index.php/WikiPathways), Pathway Commons (http://www.pathwaycommons.org/), and Panther Pathways (http://www.pantherdb.org/pathway/), also curate gene annotations, whose gene annotations are all rooted from and updated with the GOA database [38]. In order for us and readers to simply implement the interface between the proposed framework and gene annotations, we chose GOA as the source of GO terms.

In the multi-label learning scenario, a gene *g* potentially belongs to class *i* and *j* simultaneously, i.e., $g\in G_{i}\wedge g\in G_{j}$, and thus the gene *g* belongs to the positive training set and the negative training set simultaneously, i.e., $g\in G_{i}\wedge g\in (\overset{N}{\underset{j=1}{\cup }}G_{j}-G_{i})$ for the *i*-the binary model and $g\in G_{j}\wedge g\in (\overset{N}{\underset{j=1}{\cup }}G_{j}-G_{j})$ for the *j*-the binary model. In the case that the positive training set and the negative training set contain the same examples or feature vectors, we removed the negative examples or feature vectors to keep the small positive class intact.

### 2.5. L_2_-Regularized Logistic Regression

In this study, we adopted l_2_-regularized logistic regression [39] as the base classifier due to its capacity of noise resistance and fast fitting large training data with computational complexity linear to the number of training examples. Given a set of instance label pairs $\left(x_{i},y_{i}),\;i=1,2,\ldots ,l;\;x_{i}\in R^{n};\;y_{i}\in \{-1,+1\right\}$, l_2_-regularized logistic regression solves the following unconstrained optimization problem:

(1) $$\underset{\omega }{\min}\frac{1}{2}\omega^{T}\omega +C\overset{l}{\underset{i=1}{\sum }}\log(1+e^{-y_{i}\omega^{T}x_{i}})$$

where ω denotes the weight vector, C denotes the penalty parameter/regularizer, and the second term penalizes the noise/outlier fitting. The optimization of the primal problem as defined in Formula (1) is solved via its dual form:

(2) $$\begin{array}{l} \underset{\alpha }{\min}\frac{1}{2}\alpha^{T}Q\alpha +\overset{l}{\underset{i:\alpha_{i}>0}{\sum }}\alpha_{i}\log\alpha_{i}+\underset{i:\alpha_{i}<C}{\sum }\left(C-\alpha_{i}\right)\log(C-\alpha_{i})-\overset{l}{\underset{i}{\sum }}C\log C\\ subject\;to\;0\leq \alpha_{i}\leq C,i=1,\ldots ,l \end{array}$$

where $\alpha_{i}$ denotes the Lagrangian operator and $Q_{ij}=y_{i}y_{j}x_{i}^{T}x_{j}$.

### 2.6. Stratified Multi-Label Cross-Validation and Experimental Setup

As a frequently used method of model evaluation, *k*-fold cross-validation randomly split the entire training data into *k* folds of size-equal and disjoint subsets regardless of class distribution, which often leads to total absence of the minority classes from some subsets. Stratified *k*-fold cross-validation splits a dataset in a way that the class distribution of the entire dataset is approximately preserved in each fold of the subset. Empirical and theoretical studies have shown that stratified cross-validation outperforms standard cross-validation in terms of bias and variance [40]. In the multi-label learning scenario, the datasets between classes were not disjointed, so that the standard stratified cross-validation was no longer applicable. In this study, we implemented the algorithm of stratified multi-label *k*-fold cross-validation [41] to evaluate model performance.

Stratified multi-label *k*-fold cross-validation [41] achieved the goals: (1) Each fold of subset maintained the class distribution of the entire training data; (2) all the *k* folds were disjointed in the multi-label scenario; and (3) all the *k* folds were of nearly equal size. The core idea of this algorithm was to iteratively maintain each class distribution within each subset. In each iteration, the label with the fewest (but at least one) remaining examples was given a priority to be sampled and was prioritized to assign to the subset with the largest number of desired examples for this label. Interested readers are referred to [41] for details. In this study, we chose *k* = 5 for model evaluation.

The proposed framework only needs to empirically determine one hyperparameter, i.e., the regularizer *C* in Formula (1). To simplify the parameter tuning, *C* was chosen from the set $\left\{2^{-11},2^{-9},2^{-7},2^{-5},2^{-3},1,2^{3},2^{5},2^{7},2^{9},2^{11},2^{13},2^{15},2^{17}\right\}$. We chose the parameter that achieved the best *HitRate* (see the next subsection “Model evaluation metrics”).

### 2.7. Model Evaluation Metrics

The performance metrics for multi-label learning are more complicated than those for traditional supervised learning. Given an instance *i*, we proposed three metrics to measure the match degree between the true label set $L_{i}$ and the predicted label set $L_{{}_{i}}^{\prime }$, as follows.

(3) $$\begin{array}{l} HitRate(i)=\frac{\left|L_{i}\cap L_{i}^{\prime }\right|}{\left|L_{i}\right|}\\ NovelRate(i)=\frac{\left|L_{i}^{\prime }-L_{i}\right|}{\left|L_{i}^{\prime }\right|}\\ Jaccard(i)=\frac{\left|L_{i}\cap L_{i}^{\prime }\right|}{\left|L_{i}\cup L_{i}^{\prime }\right|} \end{array}$$

HitRate(*i*) denotes the rate that the true labels are correctly predicted and is used to measure the predictive ability of the proposed framework. *NovelRate(i)* denotes the rate that the predicted labels mismatch the true labels. Since the true label set (the known drugs for a target gene) is not complete, the labels beyond the true label set are not necessarily mismatches or errors. In this sense, *NovelRate(i)* can be used to measure the capability of drug discovery. The Jaccard index *Jaccard(i)* measures the overlap or consistency between the true labels and the predicted labels. Given an instance set *I* and a threshold ξ, we proposed the performance metrics for multi-label learning as follows:

(4) $$\begin{array}{l} HitRate=\frac{\left|\{i|HitRate(i)\geq \xi ,i\in I\}\right|}{\left|I\right|}\\ NovelRate=\frac{\left|\{i|NovelRate(i)\geq \xi ,i\in I\}\right|}{\left|I\right|}\\ Jaccard=\frac{\left|\{i|Jaccard(i)\geq \xi ,i\in I\}\right|}{\left|I\right|} \end{array}$$

when ξ=1, we used the metric *HitRate* to estimate how well the proposed framework correctly predicts all the true labels, and we used the metric *Jaccard* to estimate how well the predicted labels exactly match the true labels. When ξ=1|L|, the metrics were used to estimate the capability that the proposed framework at least correctly predicts one true label.

In addition, we also adopted the general-purpose performance metrics commonly-used in the multi-label learning scenario, e.g., macro-average F-measure and micro-average F-measure [42]. Assuming that there are l testing instances, $y^{i}$ denotes the true label vector of the *i*th instance and ${ў}^{i}$ denotes the predicted label vector. A set of *N* binary values, as defined in Formula (5), are used to formally define the true label and the predicted label for the *i*th instance.

(5) $$\begin{array}{l} y_{j}^{i}=\left\{ \begin{array}{l} 1{}_{}\;\;j\in L_{i}\\ 0{}_{}\;\;j\notin L_{i} \end{array}\right.,\;\;j=1,2,\ldots ,N\\ {ў}_{j}^{i}=\left\{ \begin{array}{l} 1{}_{}\;\;j\in L_{i}^{\prime }\\ 0{}_{}\;\;j\notin L_{i}^{\prime } \end{array}\right.,\;\;j=1,2,\ldots ,N \end{array}$$

For label *j*, the performance metric precision (P) and recall (R) are defined as follows.

(6) $$P=\frac{\sum_{i=1}^{l}{ў}_{j}^{i}y_{j}^{i}}{\sum_{i=1}^{l}{ў}_{j}^{i}},R=\frac{\sum_{i=1}^{l}{ў}_{j}^{i}y_{j}^{i}}{\sum_{i=1}^{l}y_{j}^{i}}$$

Since the F-measure is defined as F-measure=2×P×RP+R, the F-measure for label *j* is formally defined as follows:

(7) $$F-measure=\frac{2\sum_{i=1}^{l}{ў}_{j}^{i}y_{j}^{i}}{\sum_{i=1}^{l}{ў}_{j}^{i}+\sum_{i=1}^{l}y_{j}^{i}}$$

The macro-average F-measure is defined as the unweighted mean of the F-measures of all class labels:

(8) $$macro-average\;F-measure=\frac{1}{N}\overset{N}{\underset{j=1}{\sum }}\frac{2\sum_{i=1}^{l}{ў}_{j}^{i}y_{j}^{i}}{\sum_{i=1}^{l}{ў}_{j}^{i}+\sum_{i=1}^{l}y_{j}^{i}}$$

The micro-average F-measure considers the predictions from all instances and calculates the F-measure across all class labels as follows:

(9) $$micro-average\;F-measure=\frac{2\sum_{j=1}^{N}\sum_{i=1}^{l}{ў}_{j}^{i}y_{j}^{i}}{\sum_{j=1}^{N}(\sum_{i=1}^{l}{ў}_{j}^{i}+\sum_{i=1}^{l}y_{j}^{i})}$$

## 3. Results

### 3.1. Five-Fold Stratified Multi-Label Cross-Validation

The performance metrics for five-fold stratified multi-label cross-validation with the hyperparamer $C=2^{15}$ are illustrated in Figure 1B–D. The metrics of the F-measure for the 626 classes, calculated via Formula (7), are shown in Figure 2B, whose drug/class numberings along the horizontal axis strictly correspond to those in Figure 2A. As illustrated in Figure 2B, nearby the 300th drug/class can be viewed as a turning point, the left of which are majorly the majority classes and the right of which are majorly the minority classes. The majority classes outperform the minority classes in terms of the F-measure. The classes numbered between 200 and 300 show more encouraging performance than the other classes. The very large classes numbered below 100 contain more positive training examples but do not show high F-measure performance as expected, which is partly due to the high ratio of negatives to positives in the training data. The very small classes majorly show much lower F-measure performance, partly because these minority classes contain much fewer positive training examples. Nevertheless, the proposed framework does not show performance predominance of large classes over small classes. For a multi-label learning task with a large number of classes, it is hard to achieve well-balanced performance between classes.

The multi-label performance metrics, as defined in Formula (4), are illustrated in Figure 2C. We obtained 3.44% and 84.90% *HitRate* when the threshold was ξ=1 and ξ=1|L|, respectively, which indicates that 3.44% of genes have all the drugs correctly predicted (ξ=1) (also see Figure 2D) and 84.90% of genes have at least one drug correctly predicted (ξ=1|L|) (also see Figure 2D). For the second metric *NovelRate*, 15.1% *NovelRate* (ξ=1) indicates that 15.1% of genes have no drugs correctly predicted or all the predicted drugs are potentially new drugs, and 98.57% *NovelRate* (ξ=1|L|) indicates that 98.57% of genes have at least one novel drug discovered. For the third metric *Jaccard*, the proposed framework achieves only 0.17% Jaccard similarity (ξ=1), indicating that very few genes achieve exact matches between the true label set and the predicted label set. The more predicted drugs, the lower Jaccard similarity is achieved. The low micro-average F-measure and macro-average F-measure (see Figure 2D) also suggest that the proposed framework predicts more potentially novel drugs beyond the current true label set.

As described in the subsection “Multi-label learning framework”, the positive class is by far smaller than the negative class in each binary model, so that the binary model is potentially highly biased towards the negative class. Thus, the model is prone to yield more false negative predictions than false positive predictions. In other words, the positive predictions are presumably more credible than the negative predictions. The predicted novel labels, though mismatched with the true labels, could provide opportunities for discovery of novel drugs. To obtain more credible positive predictions, we can further choose the predictions yielded with a large probability by the binary model of l_2_-regularized logistic regression.

### 3.2. Validation against DrugBank and Matador

The proposed framework recognizes the drugs of the target genes in DrugBank [7] with 89.02% one-hit rate and 81.71% all-hit rate. The proposed framework also predicts a large fraction of novel drugs for the known target genes, with *NovelRate* equal to 100% (0.1≤ξ≤0.9) and 10.98% (ξ=1) (see Formula (4)). These results potentially indicate a risk of high false positive rate or a good capability of drug discovery. On Matador [34], the proposed framework only achieves 43.46% one-hit rate and 39.01% all-hit rate, with a larger fraction of predicted novel drugs beyond the set of known drugs. In the following subsection “Predictions for drug repurposing”, we further analyze the biological indications of the predicted novel drugs.

### 3.3. Comparison with the Existing Methods

From the methodological point of view, the existing machine learning methods commonly treat prediction of drug-target interaction as a problem of binary classification, in which the known interacting drug-target pairs are used as positive training data and the randomly sampled drug-target pairs are used as negative training data. In this proposed framework, the phenomenon that a gene is often targeted by more than one drug is naturally modelled via multi-label learning, in which each drug is treated as a class label and its target genes are used as the training data. Comparatively, the proposed framework takes several advantages over the existing binary classification methods. First, all the class-specific training data are taken from experimentally-observed drug-target interactions, except the class “non-target”, which randomly samples a very small number of drug-target pairs, accounting for a nearly negligible portion of the entire training data, whereas the existing binary classification methods, including the local models [24,25], have to randomly sample a large number of negative data, accounting for at least 50% of the entire training data. Second, the proposed framework does not limit the drug-targeted genes to the types of enzyme, ion channel, GPCR or nuclear receptor, as initially adopted by Yamanishi et al. [22] and hereinafter adopted by many other methods (see [11]). Third, the inter-class or inter-drug associations in class space are explicitly embedded into the proposed framework. Fourth, the proposed framework does not require the knowledge of drug chemical structures but only the GO knowledge of genes or gene products. Lastly, the proposed framework could better predict indirect drug-target interactions than the binary classification methods.

From the viewpoint of modeling complexity, multi-label learning with a large class space is much more complicated than binary classification. Binary classification commonly uses the area under ROC (Receiver Operating Characteristic) curve (AUC) score to measure the model performance, while multi-label learning needs to estimate the match the degree between the actual label set and the predicted label set, as defined in Formula (4). Nevertheless, we still provide the recall rate of the known drug-target interactions (DTI) for rough comparison with the binary classification methods. The proposed framework only achieves a 36.11% recall rate of five-fold cross-validation on STITCH [33], which is partly due to large class space and multi-label learning complexity. However, the proposed framework correctly recognizes 86.73% of the independent test DTIs from DrugBank [7], which indicates that the proposed framework still could generalize well to unseen examples, though it achieves low performance of cross-validation. Most of the binary classification methods only report the AUC scores that are not applicable to multi-label learning. Wen et al. [31] use drug chemical structures and protein sequences to train a deep learning model called DeepDTIs. The model achieves 82.27% recall rate of cross-validation on the known drug-target interactions, but only achieves 20.1% recall rate on the independent test data from DrugBank [7]. This result indicates that the deep learning model is potentially over-trained with a high risk of overfitting. The comparison shows that the proposed framework could generalize well in the large drug/class space without the information of drug chemical structures and target protein structures.

### 3.4. Predictions for Drug Repurposing

As analyzed above, the proposed framework shows good capability of discovering new drugs for the known target genes and new target genes for the old drugs beyond the drugs’ initial approved indications. The final class labels consist of the predicted positive labels from all the drug-specific binary models. The genes that are predicted to be targeted by novel drugs are provided in Supplementary Files S1~S3 for STITCH, Matador, and DrugBank, respectively.

File S1: Text file contains the genes from STITCH that are predicted to be targeted by novel drugs. (text format)

File S2: Text file contains the genes from Matador that are predicted to be targeted by novel drugs. (text format)

File S3: Text file contains the genes from DrugBank that are predicted to be targeted by novel drugs (text format).

Each predicted drug is assigned with a probability that indicates the confidence level of prediction. The drugs that are repurposed to other target genes are provided in Supplementary Files S4–S6 for STITCH, Matador, and DrugBank, respectively. Taking STITCH, for example, 4129 target genes are predicted to be targeted by novel drugs and 624 drugs are predicted to target novel genes. In the following sections, we take the gene, *NUDC*, and its predicted novel drug, CIDm00004156 (trideuteriomethyl methanesulfonate 1), as an example to analyze the biological and clinical implications.

File S4: Text file the drugs that are repurposed to other target genes for STITCH (text format).

File S5: Text file the drugs that are repurposed to other target genes for Matador (text format).

File S6: Text file the drugs that are repurposed to other target genes for DrugBank (text format).

### 3.5. Novel Drugs for the Gene, NUDC

**Figure 3:**
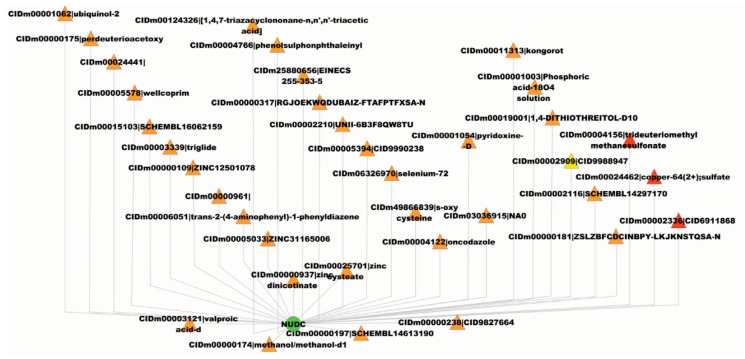
The known and predicted novel drugs that target the gene, *NUDC*. The yellow triangles denote the known drugs, the red triangles denote the predicted novel drugs and the green circle denotes the concerned gene *NUDC*.

**Figure 4:**
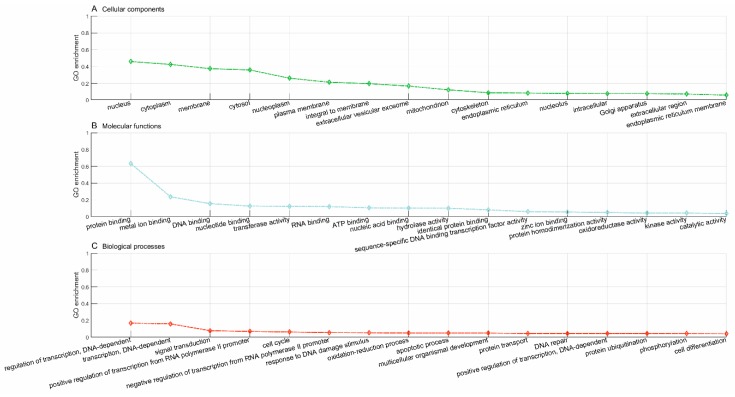
GO enrichment analysis of the known genes targeted by drug CIDm00004156.

According to STITCH [33], the gene, *NUDC*, has been known to be targeted by 33 drugs, as represented with yellow triangles in Figure 3, e.g., CIDm00011313 (kongorot), CIDm00001062 (ubiquinol-2), etc. The proposed framework predicts another three novel drugs that target the gene, *NUDC,* i.e., CIDm00024462 (copper-64(2+)) sulfate, CIDm00004156 (trideuteriomethyl methanesulfonate), and CIDm00002336 (CID6911868), as represented with red triangles in Figure 3. To gain knowledge about the novel drugs and assess the reliability of predictions, we conducted a GO enrichment analysis on the known target genes of the drugs. We took the predicted novel drug, CIDm00004156 (trideuteriomethyl methanesulfonate), for example, which has been reported to target 3679 known genes, according to STITCH [33]. We first analyzed the known genes targeted by drug CIDm00004156 and then analyzed the novel gene, *NUDC*, predicted to be targeted by drug CIDm00004156 to study the consistency. The top 15 GO terms of cellular components, molecular functions, and biological processes for the known genes targeted by drug CIDm00004156 are illustrated in Figure 4. We can see that the majority of the known target genes are subcellularly located in the nucleus (GO:0005634), cytoplasm (GO:0005737), membrane (GO:0016020), mitochondrion (GO:0005739), etc. Meanwhile, these genes majorly get involved in the biological processes of regulation of transcription DNA-dependent (GO:0006355), transcription DNA-dependent (GO:0006351), cell cycle (GO:0007049), multicellular organismal development (GO:0007275), DNA repair (GO:0006281), and cell differentiation (GO:0030154).

The gene, *NUDC*, plays indispensable roles in neurogenesis, neuronal migration, correct formation of mitotic spindles, chromosome separation during mitosis, cytokinesis, and cell proliferation (https://www.uniprot.org/uniprot/Q9Y266). The protein, NUDC, is mainly located in the nucleus (GO:0005634), cytoplasm (GO:0005737), cytoskeleton (GO:0005856), and microtubule (GO:0005874), etc., and participates in the major biological processes of the cell cycle (GO:0007049), multicellular organismal development (GO:0007275), cell differentiation (GO:0030154), cell division (GO:0051301), and developmental process (GO:0032502). The analyses show that the predicted novel gene, *NUDC*, and the known genes targeted by drug CIDm00004156 (trideuteriomethyl methanesulfonate) share highly similar patterns of subcellular localization and biological processes. In this sense, it may be safely assumed that drug CIDm00004156 could be clinically repurposed for the diseases caused by the gene, *NUDC*.

### 3.6. CIDm00004156 (Trideuteriomethyl Methanesulfonate) Drug Repurposing

**Figure 5:**
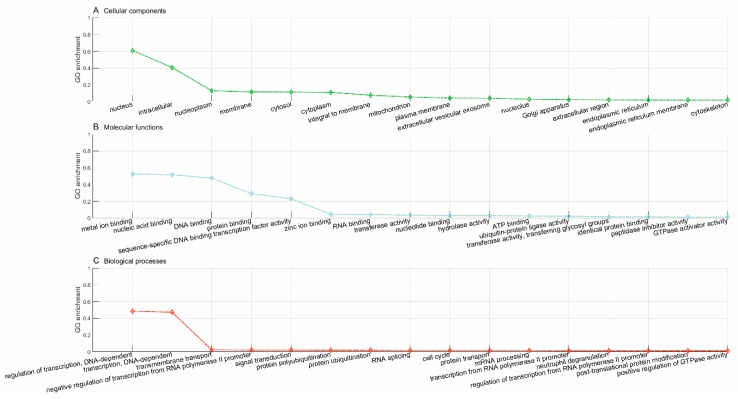
GO enrichment analysis of the predicted novel genes targeted by drug CIDm00004156.

*GO enrichment analysis*. In this study, the proposed framework predicts 544 novel genes targeted by drug CIDm00004156. We further compared the known target genes with the predicted novel genes in terms of GO enrichment. The GO enrichment analysis of the known genes targeted by drug CIDm00004156 is illustrated in Figure 4, and that of the novel genes predicted to be targeted by drug CIDm00004156 is illustrated in Figure 5. The comparison shows that the novel target genes and the known target genes demonstrate similar patterns of subcellular localization and biological processes. These results show that the predicted novel target genes are, to some extent, credible. Specifically, the novel target gene products are mainly located in the nucleus, nucleoplasm, membrane, cytoplasm, mitochondrion, and cytoskeleton, and majorly get involved in the biological processes of transcription (regulation of transcription, negative regulation of transcription from RNA polymerase II promoter, etc.), cell cycle, mRNA processing, and post-translational protein modification (protein polyubiquitination, protein ubiquitination, etc.).

**Figure 6:**
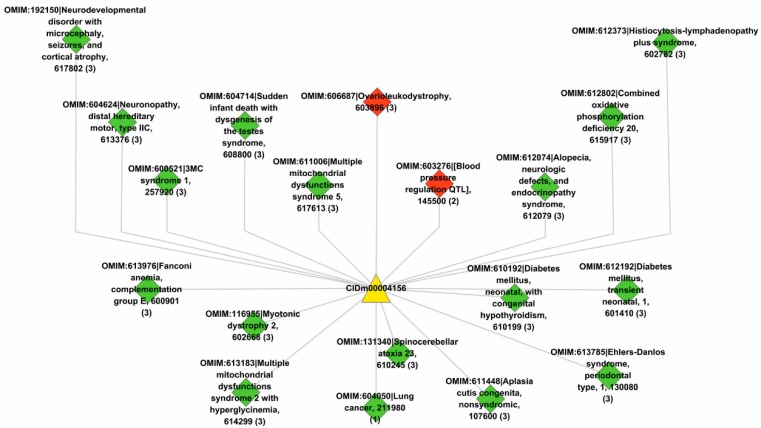
The disease phenotypes associated with drug CIDm00004156 via the predicted novel target genes. The diamond nodes in green denote phenotypes associated via at least one disease gene and the diamond nodes in red denote phenotypes associated via all the disease genes. The triangle node in yellow denotes the concerned drug CIDm00004156.

*Clinical implications*. We further mapped the predicted novel target genes of drug CIDm00004156 (trideuteriomethyl methanesulfonate) onto the OMIM database [5] to infer some potential disease phenotypes that drug CIDm00004156 is clinically applicable to. The associations between drugs and associated disease phenotypes are provided in Supplementary File S7. The disease phenotypes associated with drug CIDm00004156 via the predicted novel target genes are illustrated in Figure 6. The diamond nodes in green denote the phenotypes associated via at least one disease gene, while the diamond nodes in red denote the phenotypes associated via all the disease genes. Some of these phenotypes are closely associated with the dysfunction of the cell cycle and DNA repair, e.g., neurodevelopmental disorder with microcephaly (OMIM:192150) [43], lung cancer (OMIM:604050) [44], 3MC syndrome 1 (OMIM:600521) [45], and Fanconi anemia (OMIM:613976) [46]. These results are consistent with the evidence that drug CIDm00004156 targets the known genes that are involved in cell cycles and DNA repair (see Figure 4). In addition, some new disease phenotypes that drug CIDm00004156 is predicted to take action on, e.g., mitochondrial dysfunctions syndrome (OMIM:611006, OMIM:613183), oxidative phosphorylation deficiency (OMIM:612802), etc. (see Figure 6), are consistent with the biological processes of the known genes targeted by drug CIDm00004156, e.g., phosphorylation, oxidation-reduction process, etc. (see Figure 4).

File S7: Text file contains the associations between drugs and associated disease phenotypes (TXT).

## 4. Discussion

Identifying drug-target interactions is the primary step of drug discovery. Systems pharmacology and polypharmacology demand that a global map of many-to-many drug-target interactions be rapidly inferred to gain knowledge about drug efficacy, drug side-effects, drug targets, and drug repurposing. The existing machine learning methods generally treat prediction of drug-target interaction as a problem of binary classification, in which the known drug-target interactions are treated as the positive class and randomly sampled drug-target pairs are treated as the negative class. The major drawback of these methods is that a large number of randomly sampled accounting for over 50% of the entire training dataset are necessarily required to train a binary classifier. Such a large number of negative examples that do not come from experimental observations inevitably decrease the credibility of predictions. In this study, we model the prediction of drug-target interaction as a problem of multi-label learning by treating each drug as a class label and its target genes as the class-specific training data. As such, the inter-drug associations are explicitly modelled into the proposed framework, and all the class-specific training data come from experimental observations, except the randomly sampled “non-target” class, accounting for a nearly negligible portion of the entire training dataset.

The knowledge of drug chemical structures and protein structures convincingly increase the credibility of machine learning modeling for drug-target interaction. However, heavy dependence on this expensive information, which is not easily available in many cases, turns the merit into a second drawback to the existing methods. Even though the drug chemical structures are available, extracting a descriptor from the structures into a flat numerical vector is not an easy task and often results in information loss. In this study, the proposed framework only requires GO knowledge of the target genes, and the structural information of drugs and gene products is no longer needed. Of course, the secondary structures of proteins are now easily available or predicted, and the amino acid sequence is also used to predict drug-target interaction. With the development of techniques for encoding drug or protein structures, e.g., fingerprints and SMILES, the existing structure-based methods promise to gain a better practicability. In addition, the existing methods restrict the drug targeted genes to the types of enzyme, ion channel, GPCR, and nuclear receptor by using the drug-target interaction data initially proposed by Yamanishi et al. [22], whereas the proposed framework is applicable to all types of genes. As compared to the existing binary methods, the proposed framework does not perform so satisfactorily to correctly recognize all the class labels (drugs), due to its huge class space. It is a hard task to achieve exact matches between the true label set and the predicted label set in the multi-label learning scenario.

To reduce the class space and class imbalance, we only chose 624 drugs as class labels, and those drugs with less than 400 target genes were merged into the class “others”. As a result, the proposed framework cannot predict target genes for the drugs within the class “others”. To solve this problem, the class “others” can be further refined to train another independent multi-label learning model until the data of the remaining classes are too small to further train models.

To address the concern of class imbalance, stratified cross-validation is a commonly used policy to preserve the original class distribution in the disjoint training and test set. In the multi-label learning scenario, an example often belongs to multiple classes, rendering it complicated to preserve the disjointed relationship between training and test subsets during data partitioning of cross-validation. For this reason, we implemented the algorithm of stratified multi-label cross-validation [41] to unbiasedly estimate the proposed model.

Lastly, we used the trained model to predict new drugs for the known target genes, identify new genes for the old drugs and inferring new associations between old drugs and known disease phenotypes via the OMIM database. GO enrichment analyses show that the predicted novel target genes and the known target genes of a drug show similar patterns of subcellular localization and cellular processes. The predicted associations between drugs and diseases show that the disease phenotypes associated with identical drugs share some common molecular mechanisms. For instance, neurodevelopmental disorder with microcephaly (OMIM:192150), lung cancer (OMIM:604050), 3MC syndrome 1 (OMIM:600521), and Fanconi anemia (OMIM:613976) are all associated with dysregulation of cell cycles and DNA repair, the supporting evidences for which have been reported in recent literature [43,44,45,46]. The computational results promise to provide insights into new clinical therapies for new or old disease phenotypes and establish associations between drugs and diseases, which can be further augmented by exploring the drug-gene associations (e.g., search in the open target database (https://www.opentargets.org/)) and the gene-disease associations (e.g., search in the human protein atlas (https://www.proteinatlas.org/).

## Supplementary Materials

The following are available online at https://www.mdpi.com/1999-4923/11/9/466/s1, File S1~S7: predicted results.
